# Integrating spectral and image information for prediction of cottonseed vitality

**DOI:** 10.3389/fpls.2023.1298483

**Published:** 2023-11-13

**Authors:** Qingxu Li, Wanhuai Zhou, Hongzhou Zhang

**Affiliations:** ^1^College of Computer Science, Anhui University of Finance & Economics, Bengbu, China; ^2^College of Mechanical and Electrical Engineering, Tarim University, Alar, China

**Keywords:** cottonseed, vitality, 1D-CNN, hyperspectral, non-destructive detection

## Abstract

Cotton plays a significant role in people’s lives, and cottonseeds serve as a vital assurance for successful cotton cultivation and production. Premium-quality cottonseeds can significantly enhance the germination rate of cottonseeds, resulting in increased cotton yields. The vitality of cottonseeds is a crucial metric that reflects the quality of the seeds. However, currently, the industry lacks a non-destructive method to directly assess cottonseed vitality without compromising the integrity of the seeds. To address this challenge, this study employed a hyperspectral imaging acquisition system to gather hyperspectral data on cottonseeds. This system enables the simultaneous collection of hyperspectral data from 25 cottonseeds. This study extracted spectral and image information from the hyperspectral data of cottonseeds to predict their vitality. SG, SNV, and MSC methods were utilized to preprocess the spectral data of cottonseeds. Following this preprocessing step, feature wavelength points of the cottonseeds were extracted using SPA and CARS algorithms. Subsequently, GLCM was employed to extract texture features from images corresponding to these feature wavelength points, including attributes such as Contrast, Correlation, Energy, and Entropy. Finally, the vitality of cottonseeds was predicted using PLSR, SVR, and a self-built 1D-CNN model. For spectral data analysis, the 1D-CNN model constructed after MSC+CARS preprocessing demonstrated the highest performance, achieving a test set correlation coefficient of 0.9214 and an RMSE of 0.7017. For image data analysis, the 1D-CNN model constructed after SG+CARS preprocessing outperformed the others, yielding a test set correlation coefficient of 0.8032 and an RMSE of 0.9683. In the case of fused spectral and image data, the 1D-CNN model built after SG+SPA preprocessing displayed the best performance, attaining a test set correlation coefficient of 0.9427 and an RMSE of 0.6872. These findings highlight the effectiveness of the 1D-CNN model and the fusion of spectral and image features for cottonseed vitality prediction. This research contributes significantly to the development of automated detection devices for assessing cottonseed vitality.

## 1 Introduction

China occupies a prominent position in the realm of cotton production and processing, with its cotton planting area having surpassed 3,000 hectares over the past five years. Notably, the cotton planting area in the Xinjiang region constitutes a substantial 90% of China’s total cotton cultivation (Lu et al., 2022). The quality of cottonseed holds immense significance in the realm of cotton production, as superior-quality cottonseed exhibits a heightened germination rate, ultimately contributing to amplified cotton yields. Cottonseed quality encompasses both intrinsic and extrinsic aspects, with vitality serving as a crucial metric for gauging intrinsic quality. Elevated vitality levels are indicative of improved cottonseed germination rates (Bai et al., 2020). Currently, the management of cottonseed quality within the industry primarily relies upon manual selection. This approach, however, is limited to identifying surface defects such as breakage or mold presence (Du et al., 2023). While manual selection effectively eliminates cottonseeds with apparent cosmetic imperfections, it falls short in evaluating the inherent viability of these seeds, a factor not discernible to the naked eye. The vitality of cottonseeds holds paramount significance, as it directly impacts their potential to germinate successfully. Inadequate seed vitality precipitates suboptimal germination rates post-planting, subsequently undermining overall cotton yield and the financial returns for cotton cultivators. Consequently, there is an urgent need for techniques that can accurately ascertain the vitality of cotton seeds. To ensure the robustness of cotton production, it has become imperative to develop methodologies capable of evaluating the vitality of cottonseeds.

The research into cottonseed analysis can be classified into three main categories: cottonseed appearance assessment, variety identification, and determination of genetic modification status. Regarding appearance detection, Zhang et al. (2022) used air-coupled ultrasound with sound-to-image encoding for microcrack detection in cottonseeds, achieving a 90.7% accuracy. Wang et al. (2023) applied machine vision technology with the YOLOV5 framework to detect damaged and mold-infested cottonseeds with over 99% accuracy. Du et al. (2023) harnessed machine vision with the ResNet50 architecture for damaged cottonseed identification, reaching a 97.23% accuracy. For variety detection, Soares et al. (2016) employed near-infrared hyperspectral imaging to classify cottonseed varieties with 91.7% accuracy. Building upon this foundation, Zhu et al. (2019) introduced deep learning algorithms to further enhance cottonseed variety identification. In the context of genetically modified detection, Qin et al. (2017) employed terahertz spectroscopy for genetic modification status, achieving a 95% accuracy. Li and Shen (2020) identified noteworthy spectral peaks within the spectral ranges of 1.0~1.2 THz and 1.3~1.5 THz in genetically modified cottonseeds. Rocha et al. (2021) utilized near-infrared hyperspectral imaging to distinguish transgenic from conventional cottonseeds.While previous research has not specifically addressed cottonseed viability assessment, the studies mentioned earlier in different areas of cottonseed analysis collectively emphasize the potential use of hyperspectral technology for evaluating cottonseed quality. Hyperspectral technology excels at capturing comprehensive image data across various wavelength bands and acquiring essential optical absorption or reflection information across different wavelength ranges (Gao and Xu, 2022). The utilization of hyperspectral technology has garnered substantial traction within the realm of cotton and seed (Feng et al., 2019). For instance, Zhang et al. (2016) used it to detect foreign fibers in cotton, Li et al. (2023) to measure nitrogen levels in cotton leaves, Yan et al. (2021) to identify cotton aphid infection, and Lee et al. (2017) to detect bacterial infection in watermelon seeds. Zhou et al. (2020) achieved 89% accuracy in classifying beet seed viability, while Xu P. et al. (2022) reached 89.76% accuracy for maize seed germination. Cheng et al. (2023) applied hyperspectral detection to analyze vegetable seeds with 91% accuracy.

In summary, hyperspectral technology has been instrumental in assessing the viability of various plant seeds. Its successful implementation has been demonstrated within the realm of cotton and cottonseed cultivation. Utilizing hyperspectral technology for cottonseed vitality detection has the potential to address existing research gaps in this field. With this context in mind, a hyperspectral data acquisition system was employed to gather hyperspectral data from cottonseeds. The primary objectives encompass the acquisition of spectral and image data from cotton seeds, individualized extraction of spectral and image features inherent to cotton seeds, subsequent fusion of these extracted features, and ultimately, the construction of a predictive model for assessing the vitality of cottonseeds. This predictive model shall be devised through the utilization of both machine learning and deep learning methods.

## 2 Methods and materials

### 2.1 Sample preparation

200 seeds of the Xinluzao-57 cotton variety, sourced from Tahe Seed Company in Aral City, were chosen for this study. All cottonseeds underwent a delinting process to remove cotton fibers. The selected 200 cottonseeds were numbered. Subsequent to the comprehensive acquisition of hyperspectral data from the cottonseeds via the dedicated hyperspectral acquisition system designed for assessing the vitality of cottonseeds, all cottonseeds were earmarked for germination to ascertain their vitality. The germination experiment was executed as follows: Initially, the cottonseeds were subjected to a 15-minute scalding with boiling water. Following this, the cottonseed shells were allowed to rupture and fluff. Once this preparation was completed, the treated cottonseeds were evenly positioned within a 100 mm ×100 mm ×100 mm germination box, adhering to the pre-established sequence. A layer of loose sand, approximately 15~20 mm in thickness, was evenly distributed over the samples. Subsequently, the germination boxes were introduced into a GXZ-300A cottonseed incubator.

This process required sand grains within the box to be uniform in size, ranging from 0.05 to 0.80 mm in diameter. The sand was washed meticulously for at least 10 hours and sterilized at a high temperature of 130°C. The moisture content of the sand bed within the germination box was maintained at 80% of its saturation point. The incubation conditions were set as follows: A cycle of 12 hours for both day and night, with a daytime temperature of 27°C and light intensity at 1250 Lx. For the nighttime period, the temperature was adjusted to 20°C with no light (0 Lx). After 15 days from sowing the cottonseeds, the seedling’s height was measured using a straightedge and documented. In this study, the height of cotton seedling growth 15 days after sowing was employed as a metric for assessing the viability of cottonseeds.

### 2.2 Hyperspectral data acquisition system

**Figure 1:**
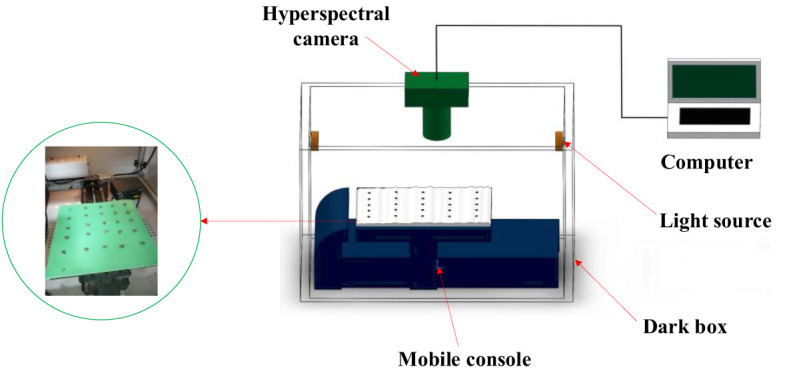
Hyperspectral data acquisition system.

The cottonseed hyperspectral data acquisition system is comprised of several essential components, illustrated in **Figure 1**, including a dark box, a hyperspectral camera, two identical tungsten halogen light sources, a mobile console, and a computer. The hyperspectral camera, specifically the Zolix HyperSIS-VNIR-CL model (manufactured by Zolix Hanguang in Beijing), exhibits a wavelength range spanning from 391 nm to 1043 nm, with a remarkable resolution of 1.25 nm. Accompanying this, the tungsten halogen light sources (manufactured by ocean optics), each possessing a power output of 50 W, operate within the wavelength range of 350 nm to 2500 nm. The dark box serves a critical purpose in averting external ambient light from interfering with the spectral camera’s operation, ensuring precision in data acquisition. The dark box is constructed from 3mm thick stainless steel with a painted surface. Concurrently, the mobile console plays a pivotal role in maneuvering the cottonseed specimens into direct alignment beneath the hyperspectral camera, facilitating optimal data capture. This orchestrated system of components collectively contributes to the meticulous acquisition of hyperspectral data from the cottonseed samples.

In the process of gathering hyperspectral data from cottonseeds, a methodical arrangement was employed. The cottonseeds were positioned in a sequential manner upon the testing plate. On each of these testing plates, a grouping of 25 cottonseeds was arranged, leading to a cumulative arrangement across 8 distinct testing plates. These plates were then situated atop the mobile console, which played a pivotal role in facilitating data collection. The acquisition parameters were configured through the SpectraSENS software interface. These parameters encompassed an exposure time of 0.20 seconds for the camera, a mobile console moving speed of 1 mm/s, and a predefined mobile console displacement range of 150 mm. The orchestrated interplay of these parameters was crucial in ensuring optimal data capture fidelity. Upon completion of the data acquisition procedure, the resulting hyperspectral data was preserved in raw file format. Each of these raw files encapsulated both spectral and image particulars associated with the 25 cottonseeds featured on the respective testing plate.

### 2.3 Dataset preparation

Sample set partitioning plays a pivotal role in influencing the efficacy and reliability of machine learning and deep learning models. The proportion of the training set to the entire dataset significantly impacts model performance, with both excessively high and overly low ratios having potential repercussions. Striking a balance is essential. A prevailing convention suggests that a ratio of 7:3 between the training set and the test set is reasonable (Shao et al., 2021). The SPXY (Sample Set Partitioning Based on Joint X-Y Distance) algorithm stands as a widely adopted approach for sample set partitioning, and at its core lies the concept of identifying similarity among samples within the feature space to allocate them to either the training set or the test set. In the context of our study, the SPXY algorithm was employed to partition a total of 200 cottonseeds into dedicated training and test sets. The training set comprised 140 cottonseeds, while the test set encompassed 60 cottonseeds.

### 2.4 Extraction of hyperspectral data

The hyperspectral camera’s imaging band range is notably narrow, rendering it susceptible to noise interference during the collection of hyperspectral images of cottonseeds. If the original hyperspectral information of the cotton seeds is utilized for analysis without proper correction, it can significantly undermine the reliability of the analysis outcomes. Consequently, a crucial step in ensuring the credibility of the analysis results is to implement a correction process on the hyperspectral data (Benelli et al., 2021). The calibration procedure is outlined as follows: Position the empty test plate atop the mobile console and capture the complete white image denoted as. Subsequently, deactivate the light sources and capture the complete black image represented as Y . By substituting these images into formula (1), the corrected hyperspectral image of the cotton seed can be obtained.


(1) $$S=\frac{O-Y}{X-Y}$$

Where, O represents the original image of the cottonseed, and S corresponds to the black and white corrected image of the cottonseed.

The corrected hyperspectral data of the cottonseeds necessitate the extraction of their spectral and image information prior to analysis and subsequent processing. In this study, the ENVI software was employed to undertake this information extraction from the rectified hyperspectral data of the cottonseeds. More specifically, this encompassed the spectral extraction of the designated region of interest (the cottonseed region within the experimental plate), as well as the extraction of images for each individual wavelength point. The hyperspectral images of the cottonseeds were obtained, with each cotton seed serving as a distinct region of interest. Notably, a total of 520 images corresponding to varying wavelength points were extracted for each individual cotton seed. Each cottonseed corresponds to a single line of spectral data.

### 2.5 Processing of spectral data

#### 2.5.1 Pretreatment for spectral data

When acquiring hyperspectral data for cottonseeds, the temperature fluctuations resulting from the heat emitted by the light source and the interference from visible light within the laboratory environment introduce additional noise to the collected data. Although the application of black-and-white correction partially mitigates this noise, its effectiveness is limited. In order to systematically diminish the detrimental influence of this noise on the subsequent data analysis processes, this study employed a combination of methodologies, including the SNV (Standard Normal Variate Transformation) algorithm, SG (Savitzky-Golay) convolutional smoothing, and MSC (Multiplicative Scatter Correction), to process the spectral data obtained from cottonseeds. Through these approaches, not only is the noise reduced, but the subsequent modeling tasks are also rendered more straightforward and user-friendly.

The SNV is primarily employed to mitigate the influence of light scattering on spectral data. This approach functions by transforming the original spectral data into standardized normal distribution variables, thereby rectifying any inherent distortions (Panda et al., 2022). The SG algorithm, rooted in the principle of least squares, operates as a polynomial smoothing technique. It leverages data points confined within a defined window to construct a polynomial curve. By doing so, this process effectively eliminates stochastic noise while preserving pertinent information intrinsic to the analyzed signals. The consequence is the enhancement of signal characteristics within the smoothed data (Yao et al., 2023). The MSC algorithm operates on the foundational premise of nullifying the ramifications of multiple scattering. This is accomplished by rectifying the spectrum of the target sample through division by a scattering reference spectrum. This corrective procedure heightens the accuracy and dependability of the spectral data. Typically, the scattering reference spectrum is an amalgamation of spectra extracted from a collection of standard samples. It is imperative that the spectral attributes of this reference align with the multiple scattering phenomena intrinsic to the target sample (Xu M. et al., 2022).

#### 2.5.2 Feature selection for spectral data

In this research, the cottonseed spectra were extracted from hyperspectral data, resulting in a data dimension of 520. However, utilizing the complete set of spectral data for modeling purposes introduces a considerable volume of redundant information, subsequently yielding suboptimal modeling outcomes. Hence, within the scope of this study, the SPA (Successive Projections Algorithm) and CARS (Competitive Adaptive Reweighted Sampling) algorithms were applied to discern the feature wavelengths within the spectral data of cottonseeds. This endeavor aimed to identify a set of pivotal wavelength positions that not only encapsulate the essence of cottonseed vitality but also expunge extraneous information.

The SPA serves as a forward feature selection technique employed to address spectral covariance quandaries. SPA operates by subjecting wavelengths to vector projection, wherein one set of wavelengths is projected onto another. Subsequently, the magnitudes of these projection vectors are juxtaposed, and the wavelength boasting the most substantial projection vector is chosen. This preliminary selection serves as the basis for further feature wavelength selection, facilitated through a corrective model. SPA effectively assembles a subset of variables that minimizes both redundancy and covariance, thus optimizing information content (Tang et al., 2018). The CARS algorithm employs a strategy of adaptive reweighted sampling to pinpoint wavelength positions characterized by substantial absolute regression coefficients within the partial least squares model. This approach involves eliminating wavelength positions with minor weights and leveraging cross-validation to identify a subset with the least cross-validated mean squared deviation values. Consequently, this methodology streamlines the search for an optimal amalgamation of variables, enhancing overall efficiency (Lin et al., 2019).

### 2.6 Extraction of image features

**Figure 2:**
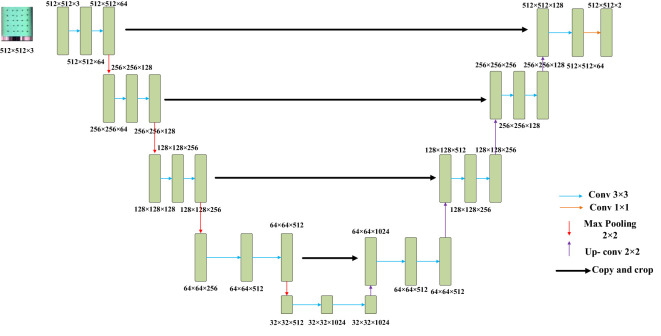
U-Net.

Two prevalent techniques for hyperspectral image analysis deserve mention: Firstly, the conversion of hyperspectral imagery into a color representation allows for the extraction of features like chromatic attributes and color-based morphological characteristics. The second approach involves decomposing the high-dimensional image data into individual single-channel images. Subsequently, the texture intricacies within these single-channel images are subjected to extraction. Given the subtle differentiations in color and morphological attributes within cottonseed images, this study opted to harness texture features for prognosticating cottonseed vitality. However, it’s important to note that each individual cottonseed image in this study comprises 25 distinct cottonseeds, thus necessitating individual segmentation for accurate analysis. The segmentation task was executed using the U-Net architecture, which comprises a compression path and an expansion path. Within the compression path, four blocks were incorporated, each consisting of three convolutions and a max pooling downsampling operation. The number of feature maps was consistently doubled post each downsampling operation. Correspondingly, the expansion path, also comprised of four blocks, initiated with three successive convolutional downsampling operations, succeeded by an additional Max Pooling downsampling step. In each block, the feature map’s size was magnified twofold, subsequently halving its count through inverse convolution. This augmented map was then amalgamated with the feature map from the symmetrical compression path on the left, as shown in **Figure 2** (Beeche et al., 2022).

Upon accomplishing the segmentation of individual cottonseeds, the ensuing step involves the extraction of texture features for each isolated cottonseed. This process entails the application of the gray-scale co-occurrence matrix, grounded in the concept that each pixel’s frequency of occurrence within a specific range of neighboring pixels, all possessing identical gray levels, is tallied. The resultant counts are subsequently employed as elements within the Gray-Level Co-occurrence Matrix (GLCM) corresponding to the given pixel (Hussain et al., 2022). The mathematical formulation for its implementation is as follows:


(2) G(i,j)=∑p(i,j)

This formula, *G(i, j)* represents the frequency of co-occurrence of a pixel possessing a gray level i alongside a pixel at a distance j with the same gray level. Meanwhile, p(i,j) signifies the normalized GLCM, effectively capturing the proportional distribution of such co-occurring instances.

The features extracted from the GLCM encompass several fundamental attributes: Contrast (This descriptor encapsulates the disparity between distinct gray levels within the image texture, thereby delineating texture contrasts); Correlation (By characterizing the interconnectedness of pixel gray levels in the image texture, correlation offers insights into the interrelationships within the texture); Energy (Reflecting the extent of textural intricacy, energy gauges the presence and intensity of detailed textural patterns within the image); Entropy (This facet captures the intricacy and ambiguity present within the image texture, signifying its level of uncertainty and complexity). In this study, individual cottonseed comprises 520 images spanning various spectral bands. Each of these images is associated with four distinctive metrics for texture attributes. Consequently, a cumulative total of 2080 texture features are derived for each cottonseed.

### 2.7 Modeling methods

#### 2.7.1 PLSR and SVR

Within this study, the prognostication of cottonseed vitality was pursued through the application of two distinct regression models: Partial Least Squares Regression (PLSR) and Support Vector Regression (SVR). PLSR stands as a statistical analytical technique primarily employed for establishing regression connections among multiple variables. This method finds frequent application in addressing regression challenges arising from high-dimensional data and multicollinearity. PLSR employs a decomposition strategy, breaking down both the predictor and response variables into latent variables. Subsequently, it establishes a linear association between these latent variables, achieved by minimizing the covariance existing between them (Cheng and Sun, 2017). At the core of SVR lies the principle of minimizing the dissonance between predicted and actual outcomes, achieved by determining an optimal hyperplane that seamlessly maps input data to corresponding output data (Sun et al., 2020). This procedural journey encompasses the following steps:

1. Input data undergoes the transformation into a feature space with an elevated dimensionality.

2. Within this augmented feature space, a foundational hyperplane is erected, serving as the bedrock for prediction-making and facilitating the regression endeavor.

3. The procedure includes the identification of support vector data points residing in close proximity to the hyperplane within the feature space. These vectors play a pivotal role in establishing the hyperplane’s placement.

4. The optimization of hyperplane parameters is accomplished by minimizing a designated objective function.

#### 2.7.2 1D-CNN

**Figure 3:**
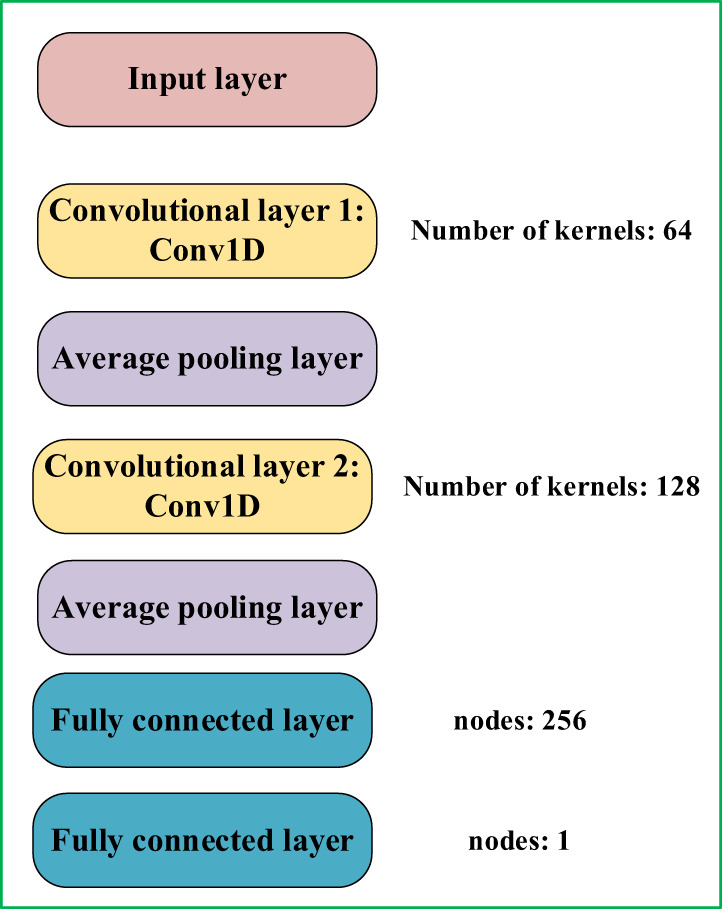
1D-CNN for Cottonseed.

Convolutional neural networks exhibit robust feature extraction capabilities and have demonstrated notable achievements in both classification and regression tasks (Guo et al., 2022). Given the distinctive characteristics intrinsic to cottonseed spectral and image texture data, this research employed a one-dimensional convolutional neural network (1D-CNN) to prognosticate the vitality of cottonseeds. To enhance the adaptability of the 1D-CNN model for cottonseed vitality prediction, a tailored 7-layer architecture was constructed, depicted in **Figure 3**. This architecture encompasses two sequential 1D convolutional layers, supplemented by two average pooling layers and two fully connected layers. The initial 1D convolutional layer incorporates 64 convolutional kernels, while the second layer integrates 128 convolutional kernels. These convolutional layers are pivotal in extracting essential characteristics from the cottonseed data. The incorporation of average pooling layers expedites model convergence and serves as a preventive measure against overfitting. Within the fully connected layers, the first layer accommodates 256 neurons, while the subsequent layer is composed of a single neuron, which specifically signifies cottonseed vitality.

The selection of a suitable loss function profoundly influences model performance, as it guides the continual refinement of network parameters throughout the training phase by quantifying the disparity between predicted and actual values. By acting as a yardstick for this discrepancy, the choice of an appropriate loss function holds the potential to expedite convergence while enhancing model efficacy. Within this study, the mean square error function was adopted to quantify the disparity between predicted and actual cottonseed vitality values. The computational formulation for this function is as follows:


(3) $$MSE=\frac{\sum_{i=1}^{n}{\left(\hat{y}-y\right)}^{2}}{n}$$

Where $\hat{y}$ signifies the predicted cottonseed vitality value, y denotes the actual cottonseed vitality value. n corresponds to the count of cottonseed samples.

### 2.8 Performance evaluation of models

In the process of employing the U-Net for cottonseed segmentation, this study evaluates the model’s segmentation performance using two widely employed metrics in image semantic segmentation tasks: Pixel Accuracy (PA) and Mean Intersection Over Union (MIoU). These metrics are applied to compare and analyze the model’s semantic segmentation outcomes against manually annotated cottonseed images. The formulas for both metrics are presented below:


(4) $$PA=\frac{\sum_{i=0}^{N-1}n_{ii}}{\sum_{i=0}^{N-1}\sum_{j=0}^{N-1}n_{ij}}\times 100\%$$

(5) $$MIoU=\frac{1}{N+1}\sum_{j=0}^{N-1}\frac{n_{ii}}{\sum_{j=0}^{N-1}n_{ij}+\sum_{j=0}^{N-1}n_{ji}-n_{ii}}\times 100\%$$

In these formulas, N represents the count of semantic categories, which, in this study, is set to 2. $n_{ii}$ denotes the tally of accurate pixels corresponding to category i semantics. Similarly, $n_{ij}$ signifies the count of pixel points in which category i semantics is erroneously identified as category j , while $n_{ji}$ indicates the count of pixel points where category j semantics is erroneously identified as category i .

The assessment metrics for the regression model encompass the correlation coefficient and the root mean square error. Generally, a model’s predictive efficacy is deemed higher when the correlation coefficient approaches 1 and the root mean square error approaches 0. The computation of these metrics is outlined below:


(6) $$R=\frac{\sqrt{\sum_{i=1}^{n}{\left({\hat{y}}_{i}-y_{i}\right)}^{2}}}{\sqrt{\sum_{i=1}^{n}{\left({\hat{y}}_{i}-y_{mean}\right)}^{2}}}$$

(7) $$RMSE=\sqrt{\frac{1}{n}\sum_{i=1}^{n}{\left({\hat{y}}_{i}-y_{i}\right)}^{2}}$$

Where, n denotes the count of samples within the dataset. ${\hat{y}}_{i}$ signifies the predicted value for the “ith” sample, $y_{i}$ represents the actual value of the same “ith” sample. Additionally, $y_{mean}$ stands for the mean value computed from the actual values across all samples encompassed by the dataset.

## 3 Results and discussion

### 3.1 Analysis results of spectral data

#### 3.1.1 Sensitive band analysis of cottonseed

**Figure 4:**
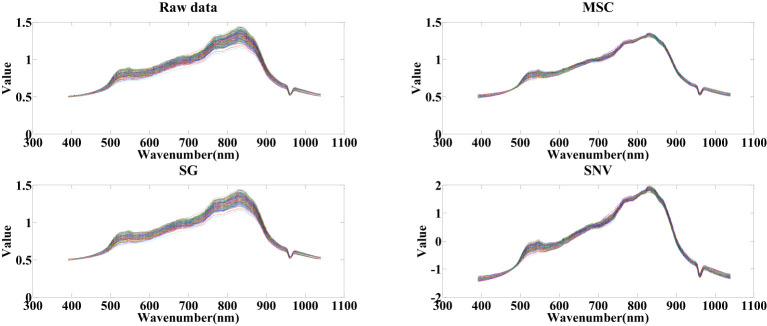
Spectral data after pretreated.

After extracting hyperspectral data, a dataset comprising 200 cottonseed samples was compiled, encompassing both spectral and image data. This section is dedicated exclusively to harnessing spectral data for predicting cottonseed vitality. Before selecting feature wavelengths, the cottonseed spectral data undergoes pretreatment via three distinct algorithms: SNV, MSC, and SG. These algorithms are employed to counteract the effects of noise and scattering on the modeling outcomes. Illustrated in **Figure 4**, observations discern that following SG pretreatment, the distribution of cottonseed spectral data exhibits similarities to the original distribution, albeit with heightened smoothness. With MSC pretreatment, the distribution of cottonseed spectral data becomes more concentrated in contrast to the original dataset. Conversely, the values of cottonseed spectral data undergo modification after SNV pretreatment, resulting in a distribution akin to that achieved through MSC pretreatment.

**Figure 5:**
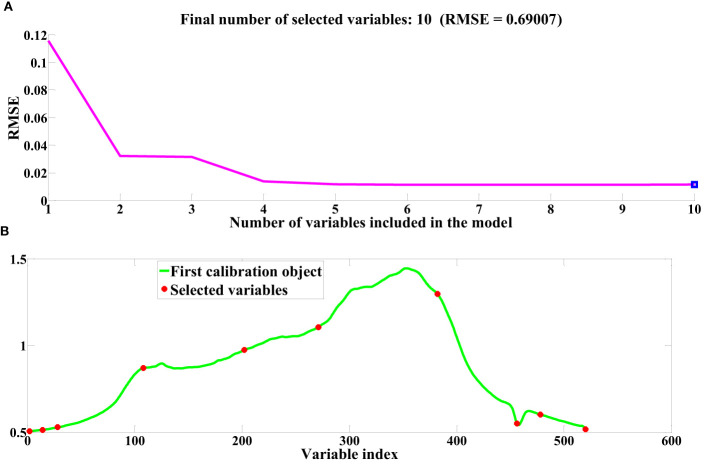
Feature wavelength selection based on SPA. **(A)** RMSE **(B)** Index of selected variables.

Following the pretreatment of cottonseed spectral data, the SPA and CARS algorithms were employed to select feature wavelengths. This procedure aimed to identify essential sets of wavelength points that effectively encapsulate cottonseed vitality. The progression of feature wavelength selection via the SPA is depicted in **Figure 5**, utilizing the cottonseed spectral data following SG pretreatment as an illustrative example. The fundamental tenet of the SPA algorithm for feature wavelength selection in cottonseed spectral data is rooted in the minimization of the root mean square error (RMSE), as depicted in **Figure 5A**. Notably, the RMSE reaches its minimum value when 10 features are chosen. The specific feature wavelengths selected in this process are illustrated in **Figure 5B**. The selection of feature wavelength points following MSC and SNV pretreatment mirrored that of SG. Ultimately, we identified 10 characteristic wavelength points after SG preprocessing, 8 after MSC, and 6 after SNV, distributed across both the visible and near-infrared wavelength ranges.

**Figure 6:**
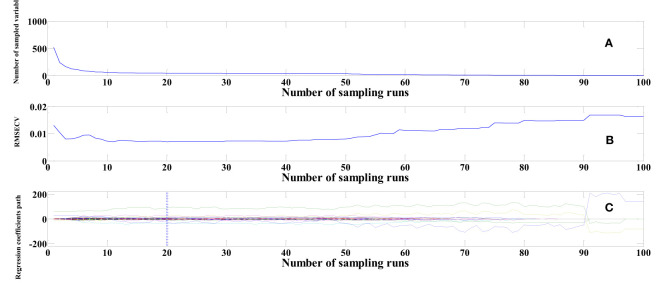
Feature wavelengths selection based on CARS. **(A)** Number of sampled variables **(B)** RMSECV **(C)** Regression coefficients path.

To elucidate the process of extracting feature wavelengths using the CARS algorithm, the same SG-pretreated cottonseed spectral data serves as an illustrative example. This study implements 100 Monte Carlo sampling iterations and employs a 5-fold cross-validation approach. As evidenced in **Figure 6A**, the count of selected variables gradually diminishes as the number of sampling iterations progresses. **Figure 6B** reveals the behavior of the Root Mean Square Error of Cross Validation (RMSECV), depicting a gradual decline followed by an eventual increase. The decrement in RMSECV indicates the removal of extraneous information from the cottonseed spectral data, while the subsequent rise in RMSECV suggests the elimination of vital information. The point at which RMSECV reaches its minimum value is accompanied by the presentation of regression coefficients for each variable along the vertical line in **Figure 6C**. At this juncture, the number of sampling iterations is recorded as 20. The choice of feature wavelengths post MSC and SNV pretreatment closely resembled that of SG. Ultimately, we identified 45 feature wavelength points after SG pretreatment, 64 after MSC, and 53 after SNV, distributed across both the visible and near-infrared wavelength bands.

#### 3.1.2 Regression prediction based on PLSR, SVR

Following the identification of feature wavelengths capable of indicating the vitality of cotton seeds, we employed PLSR and SVR techniques to formulate a robust predictive model for cotton seed vitality assessment. Within the framework of this investigation, three principal components were chosen for PLSR modeling, while the radial basis function emerged as the optimal choice for SVR analysis. Detailed outcomes of these models are presented in **Table 1**. Among the discriminant models for cottonseed vitality developed through PLSR, the model constructed utilizing the synergistic integration of SG pretreatment and SPA treatment exhibited unparalleled predictive prowess. This model showcased exceptional predictive efficacy, boasting a correlation coefficient of 0.8709 and an impressively low RMSE of 0.8027 when evaluated against the test dataset. In contrast, the model generated by applying SNV pretreatment in conjunction with SPA treatment demonstrated a comparatively suboptimal predictive performance. This model was characterized by a correlation coefficient of 0.6970 and a relatively higher RMSE of 1.0685 when scrutinized against the same test dataset. Amidst the suite of SVR models crafted, the model fashioned through the amalgamation of SG pretreatment and SPA treatment emerged as the apex performer. This exemplary model exhibited a correlation coefficient of 0.8917 and an RMSE of 0.7435 when subjected to evaluation against the designated test dataset. In contrast, the model devised by employing SNV pretreatment in conjunction with SPA treatment displayed comparatively less favorable performance metrics. Specifically, this model registered a correlation coefficient of 0.8064 and an RMSE of 0.9606 when assessed against the same comprehensive test dataset.

**Table 1:** The results of PLSR, SVR.

Model	Pre-Processing	VN	R_C_	RMSE_C_	R_P_	RMSE_P_
PLSR	SG + SPA	10	0.9285	0. 6972	0.8709	0.8027
	SG + CARS	45	0.9456	0.6783	0.8517	0.8645
	MSC + SPA	6	0.9157	0.7137	0.8461	0.9014
	MSC + CARS	64	0.9582	0.6624	0.8583	0.8352
	SNV + SPA	8	0.7159	1.0216	0.6970	1.0685
	SG + SPA	10	0.9637	0.6521	0.8917	0.7435
SVR	SG + SPA	10	0.9637	0.6521	0.8917	0.7435
	SG + CARS	45	0.9563	0.6689	0.8422	0.9160
	MSC + SPA	6	0.9088	0.7356	0.8632	0.8274
	MSC + CARS	64	0.9257	0.6997	0.8816	0.7952
	SNV + SPA	8	0.8562	0.8456	0.8064	0.9606
	SNV + CARS	53	0.9146	0.7204	0.8347	0.9231

R_C_, Correlation coefficient of the training set; RMSE_C_, RMSE of the training set; R_P_, Correlation coefficient of the test set; RMSE_P_, RMSE of the test set; VN, number of variables.

#### 3.1.3 Regression prediction based on 1D-CNN

**Figure 7:**
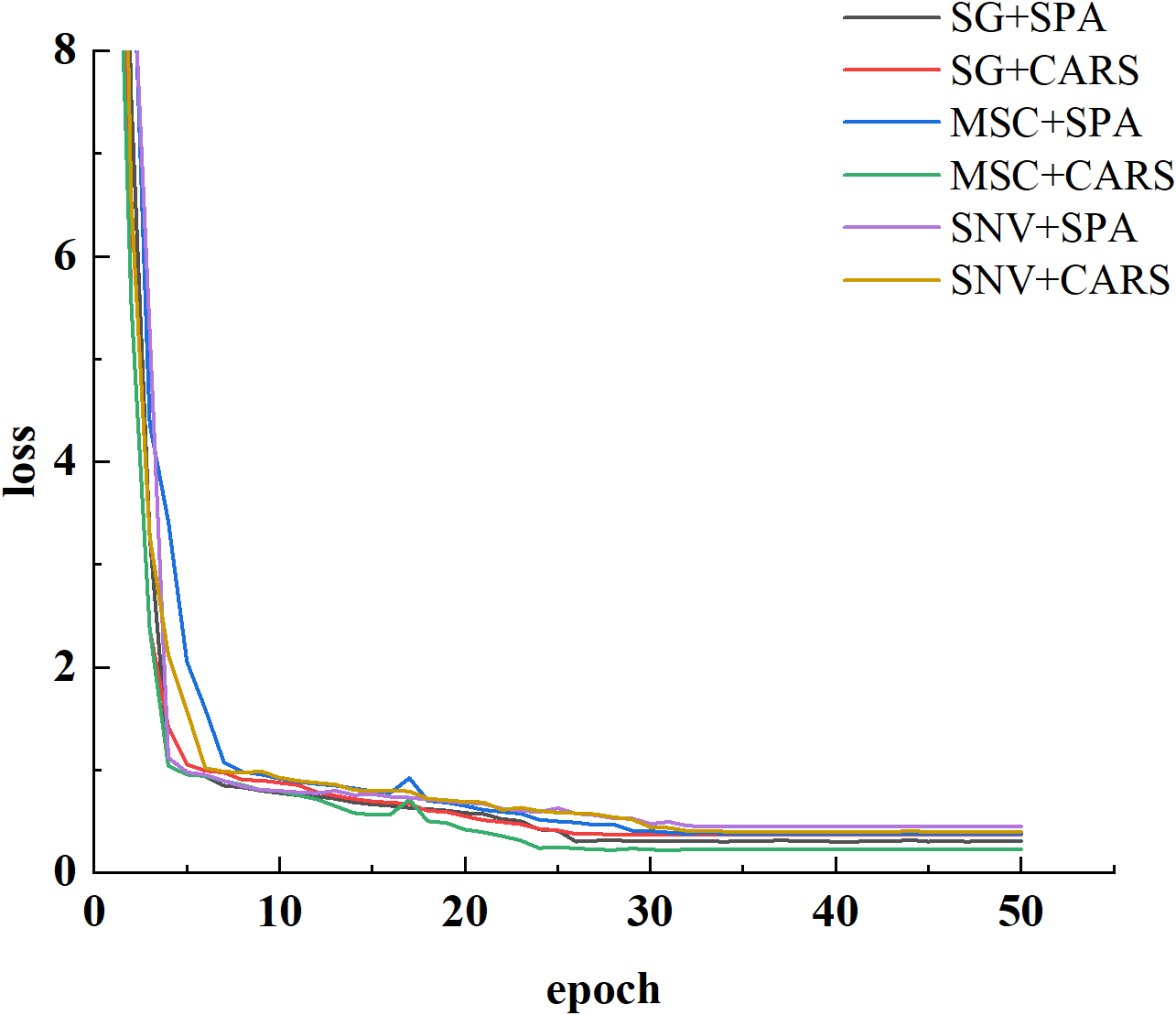
Loss curves.

In this study, we employed a 1D-CNN to construct a robust predictive model for assessing cottonseed vitality. The model training was executed within a hardware framework comprising an i9-12900K CPU, NVIDIA GeForce RTX 3090Ti GPU, and operating on the Windows 10 platform. The software environment encompassed Pytorch 1.12 coupled with CUDA 11.7 for efficient computational acceleration. Network parameter optimization was achieved through the SGD optimizer, with an initial learning rate established at 0.0001 and a predefined maximum training iteration of 50. Notably, a batch size of 4 was employed during the training process. The preprocessed cottonseed data, following pretreatment and feature wavelength selection, were harnessed as inputs for the 1D-CNN. The dynamics of network training reflected through the progression of loss, are visually illustrated in **Figure 7**. Evidently, following 20 epochs of training, the loss values across the spectrum of six distinct treatments have substantially converged to a low magnitude. This convergence underscores the attainment of model stability. Notably, the model attained its lowest loss value subsequent to the application of MSC in conjunction with CARS preprocessing. Conversely, the highest loss value was observed following the utilization of SNV pretreatment accompanied by SPA treatment.

The modeling results for the 1D-CNN are summarized in **Table 2**. It is evident that the model constructed after applying MSC+CARS preprocessing exhibits the most outstanding performance in predicting the vitality of cottonseed. This is supported by a test set correlation coefficient of 0.9214 and an RMSE of 0.7017. Conversely, the model developed after employing SNV+SPA preprocessing demonstrates the poorest performance, as indicated by a test set correlation coefficient of 0.8215 and an RMSE of 0.9451. These findings are also consistent with the results obtained during the training of the 1D-CNN model, where the convergence of loss values further validates the observed trends.

**Table 2:** The results of 1D-CNN.

Pre-Processing	VN	R_C_	RMSE_C_	R_P_	RMSE_P_
SG + SPA	10	0.9532	0.6704	0.9047	0.7386
SG + CARS	45	0.9459	0.6783	0.8637	0.8269
MSC + SPA	6	0.9478	0.6724	0.8503	0.8715
MSC + CARS	64	0.9728	0.3927	0.9214	0.7017
SNV + SPA	8	0.9259	0.6990	0.8215	0.9451
SNV + CARS	53	0.9644	0.6497	0.8459	0.9002

R_C_, Correlation coefficient of the training set; RMSE_C_, RMSE of the training set; R_P_, Correlation coefficient of the test set; RMSE_P_, RMSE of the test set; VN, number of variables.

### 3.2 Analysis results of image data

#### 3.2.1 Cottonseed segmentation

**Figure 8:**
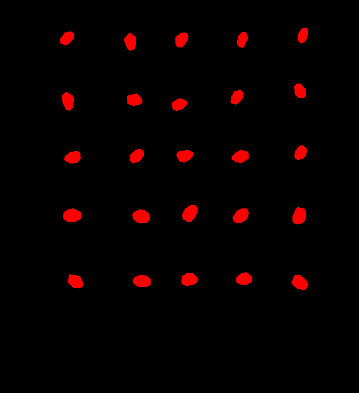
Cottonseed segmentation results.

In this study, we employed the Labelme annotation tool to annotate cotton seeds from six test plates. Subsequently, the cottonseed images were segmented using the U-Net network. Given the limited number of cotton seed images available for this study, we initialized the U-Net network with pre-trained weights from the COCO Stuff dataset. The hardware and software platforms utilized for training the U-Net network included an Intel i9-12900K CPU, NVIDIA GeForce RTX 3090Ti GPU, PaddlePaddle 2.5, and CUDA 11.7. The segmentation results of the model are presented in **Figure 8**. It is evident that U-Net achieves results for cottonseed segmentation, with a PA of 97.88% and an MIoU of 88.53%. Moreover, the model demonstrates efficient performance with a single-image detection time of 320ms. These findings indicate a superior segmentation capability that fully meets the segmentation requirements for this study.

#### 3.2.2 Texture feature extraction from cottonseeds

**Figure 9:**
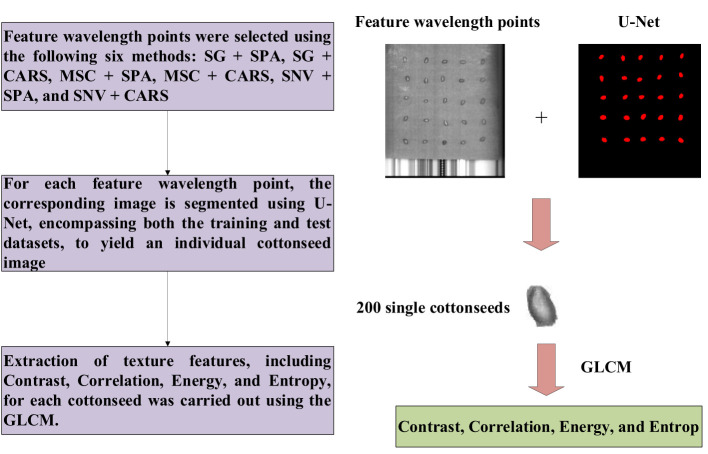
Texture feature extraction from cottonseeds.

The segmentation of cottonseed images corresponding to feature wavelength points selected by six distinct processing methods was conducted using the pre-trained U-Net network described earlier. Following the completion of segmentation, four texture features (Contrast, Correlation, Energy, Entropy) were individually extracted for each cottonseed using the GLCM. For instance, when considering the feature wavelength point of 711nm for the cottonseed, the U-Net network was utilized to segment the corresponding image at 711nm. Subsequently, four texture features were extracted for each segmented cottonseed, as illustrated in **Figure 9**. After completing the extraction of texture features from cottonseeds, we employed PLSR, SVR, and 1D-CNN to construct prediction models for cottonseed vitality. The results are presented in **Table 3**. For PLSR, the images corresponding to the feature wavelength points selected with SNV+SPA exhibited the best performance in predicting cottonseed vitality, achieving a test set correlation coefficient of 0.7743 and an RMSE of 0.9936. Similarly, for SVR, the SNV+SPA preprocessing outperformed others, yielding a test set correlation coefficient of 0.7524 and an RMSE of 1.0184. On the other hand, when employing 1D-CNN, the SG+CARS preprocessing demonstrated superior performance in predicting cottonseed vitality, with a test set correlation coefficient of 0.8032 and an RMSE of 0.9683.

**Table 3:** The results of PLSR, SVR and 1D-CNN.

Model	Pre-Processing	VN	R_C_	RMSE_C_	R_P_	RMSE_P_
PLSR	SG + SPA	40	0.8237	0.9402	0.7539	1.0164
	SG + CARS	180	0.8069	0.9601	0.6753	1.2767
	MSC + SPA	24	0.7958	0.9968	0.6568	1.3984
	MSC + CARS	256	0.7012	1.0482	0.5914	1.6323
	SNV + SPA	32	0.8369	0.9201	0.7743	0.9936
	SNV + CARS	212	0.8058	0.9678	0.6288	1.5108
SVR	SG + SPA	40	0.8491	0.8967	0.7392	1.1573
	SG + CARS	180	0.8236	0.9389	0.6647	1.3372
	MSC + SPA	24	0.7792	1.0127	0.6329	1.4736
	MSC + CARS	256	0.6218	1.5789	0.4788	1.7729
	SNV + SPA	32	0.7965	0.9693	0.7524	1.0184
	SNV + CARS	212	0.7284	1.2635	0.6342	1.4628
1D-CNN	SG + SPA	40	0.8316	0.9179	0.7906	0.9755
	SG + CARS	180	0.8527	0.8566	0.8032	0.9683
	MSC + SPA	24	0.8033	0.9705	0.6734	1.2982
	MSC + CARS	256	0.8939	0.7493	0.5983	1.6024
	SNV + SPA	32	0.7748	1.0194	0.7632	0.9983
	SNV + CARS	212	0.8247	0.9397	0.6346	1.4623

R_C_, Correlation coefficient of the training set; RMSE_C_, RMSE of the training set; R_P_, Correlation coefficient of the test set; RMSE_P_, RMSE of the test set; VN, number of variables.

### 3.3 Analysis results of fused spectral and image data

In hyperspectral data analysis, the fusion of image and spectral data typically involves two methods: one is the direct fusion of spectral feature wavelengths with all image features, and the other is the fusion of a feature wavelength point with its corresponding image features. In this study, the first method results in image features with 2080 dimensions, which can potentially lead to overfitting of the model if applied directly. Hence, we integrated the extracted spectral feature wavelength point data with the corresponding image texture features. Following feature fusion, this study employed PLSR, SVR, and 1D-CNN to construct prediction models, and the outcomes are presented in **Table 4**. From the tables, it is evident that all three models constructed after the SG+SPA preprocessing exhibited the highest performance in predicting cottonseed vitality. They achieved a test set correlation coefficient of 0.8892, 0.9056, and 0.9427, with corresponding RMSE of 0.7904, 0.7349, and 0.6872 for PLSR, SVR, and 1D-CNN, respectively. Notably, this performance improvement was notable when compared to the utilization of spectral data or image texture features in isolation.

**Table 4:** The results of PLSR, SVR and 1D-CNN.

Model	Pre-Processing	VN	R_C_	RMSE_C_	R_P_	RMSE_P_
PLSR	SG + SPA	50	0.9396	0. 6851	0.8892	0.7904
	SG + CARS	225	0.9782	0.3643	0.8539	0.8612
	MSC + SPA	30	0.9214	0.7011	0.8607	0.8301
	MSC + CARS	320	0.9805	0.3428	0.8596	0.8317
	SNV + SPA	40	0.8571	0.8439	0.8033	0.9608
	SNV + CARS	265	0.9065	0.7392	0.8104	0.9563
SVR	SG + SPA	50	0.9688	0.6493	0.9056	0.7349
	SG + CARS	225	0.9806	0.3425	0.8512	0.8693
	MSC + SPA	30	0.9329	0.6896	0.8704	0.8167
	MSC + CARS	320	0.9863	0.3209	0.8805	0.7973
	SNV + SPA	40	0.9017	0.7420	0.8439	0.9016
	SNV + CARS	265	0.9733	0.3922	0.8531	0.8688
1D-CNN	SG + SPA	50	0.9667	0.6415	0.9427	0.6872
	SG + CARS	225	0.9704	0.3729	0.9069	0.7356
	MSC + SPA	30	0.9376	0.6802	0.8674	0.8682
	MSC + CARS	320	0.9865	0.3368	0.9295	0.6987
	SNV + SPA	40	0.9582	0.6690	0.8544	0.8613
	SNV + CARS	265	0.9732	0.3704	0.8629	0.8701

R_C_, Correlation coefficient of the training set; RMSE_C_, RMSE of the training set; R_P_, Correlation coefficient of the test set; RMSE_P_, RMSE of the test set; VN, number of variables.

### 3.4 Comparison of optimal models for spectral, image, and spectral-image fusion

**Figure 10:**
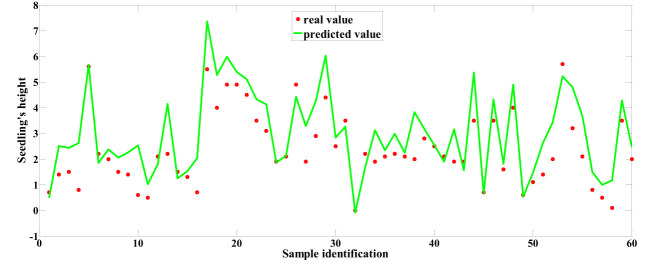
Predicted values based on spectral data.

**Figure 11:**
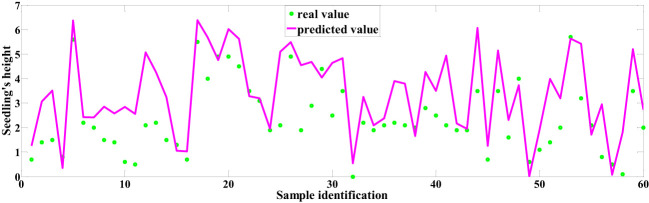
Predicted values based on image data.

**Figure 12:**
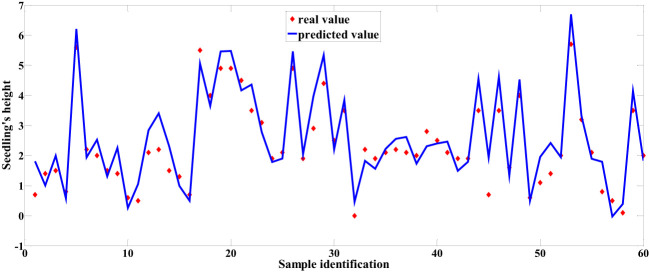
Predicted values based on fused spectral and image data.

Among the predictive models for cottonseed vitality based on spectral data, the 1D-CNN model, established after applying MSC+CARS preprocessing, demonstrated the highest performance. It achieved a test set correlation coefficient of 0.9214 and an RMSE of 0.7017, as illustrated in **Figure 10**. In the case of predictive models for cottonseed vitality constructed using hyperspectral image data, the 1D-CNN model, developed following SG+CARS preprocessing, exhibited the best performance, with a test set correlation coefficient of 0.8032 and an RMSE of 0.9683, as depicted in **Figure 11**. Furthermore, among the models that integrated both spectral and image data, the 1D-CNN model, established after SG+SPA preprocessing, outperformed others, boasting a test set correlation coefficient of 0.9427 and an RMSE of 0.6872, as illustrated in **Figure 12**. The optimal performance of the cottonseed vitality prediction model, incorporating both spectral and image features, is evident.

### 3.5 Discussion

To address the challenge of effectively assessing the vitality of cottonseeds during the cotton cultivation process, this study employed hyperspectral technology to develop a data acquisition system dedicated to cotton seeds. Subsequently, prediction models for cottonseed vitality are established using spectral data, image data, and fused spectral-image data. The modeling techniques encompass both machine learning and deep learning methodologies. Notably, while there are existing studies focusing on various qualities of cottonseed, such as Wang et al. (2023) achieving a 99% accuracy in detecting broken and mold-infested cottonseeds using YOLOV5, and Du et al. (2023) achieving a 97.23% accuracy in detecting broken cottonseeds, and also research on the identification of genetically modified cottonseeds (Li et al., 2020; Qin et al., 2017), no prior research has addressed cottonseed vitality detection. This study fills this research gap and additionally compares the application of hyperspectral detection for assessing the vitality of other plant seeds, such as vegetable seeds (Cheng et al., 2023), maize seeds (Xu P. et al., 2022), and beet seeds (Zhou et al., 2020). Furthermore, we successfully maintained consistency in achieving predictions even with thicker and harder seed shells, as demonstrated in cottonseed vitality predictions.

## 4 Conclusions

In this study, hyperspectral data of cotton seeds was collected, and we conducted separate extractions of spectral data and corresponding image data from different bands. The identification of feature wavelength points for cottonseeds was achieved through a combination of SG, SNV, and MSC pretreatment algorithms in conjunction with SPA and CARS techniques. Subsequently, we developed distinct models for predicting the vitality of cottonseeds using the following datasets: spectral data alone, image data alone, and a fused dataset combining spectral and image data. In terms of spectral data analysis, the 1D-CNN model, constructed following MSC+CARS preprocessing, demonstrated the highest performance, boasting a test set correlation coefficient of 0.9214 and an RMSE of 0.7017. Turning to image data, the U-Net network exhibited remarkable capabilities with a PA of 97.88% and an MIoU of 88.53%, ensuring precise cottonseed segmentation. Leveraging the four texture features extracted from the images, corresponding to the wavelength points of interest, the 1D-CNN model, established after SG+CARS preprocessing, yielded the most effective results for predicting cottonseed vitality, attaining a test set correlation coefficient of 0.8032 and an RMSE of 0.9683. For fused spectral and image data, the model’s optimal performance was observed after SG+SPA preprocessing, delivering a test set correlation coefficient of 0.9427 and an RMSE of 0.6872. Image information primarily portrays the external attributes of cottonseeds, whereas spectral data can reveal crucial insights about the internal composition of the cottonseed. The vitality of cottonseeds is influenced by both the shell and kernel. Therefore, the fusion of spectral and image information leads to improved cottonseed vitality prediction. Furthermore, it’s worth noting that the 1D-CNN model’s performance in this study surpassed that of SVR and PLSR, indicating its suitability for cottonseed vitality prediction. These findings hold significant promise in providing crucial technical support for the development of future automated cottonseed vitality detection devices.

## Data availability statement

The datasets presented in this article are not readily available. This study’s data will continue to be used in subsequent research. The original data cannot be provided. Requests to access the datasets should be directed to 120220059@aufe.edu.cn.

## Author contributions

QL: Conceptualization, Data curation, Formal Analysis, Investigation, Methodology, Software, Validation, Visualization, Writing – original draft. WZ: Writing – review & editing. HZ: Funding acquisition, Investigation, Project administration, Resources, Writing – review & editing.
